# Urethral catheters: can we reduce use?

**DOI:** 10.1186/1471-2490-11-10

**Published:** 2011-05-23

**Authors:** Pieter J van den Broek, Jan C Wille, Birgit HB van Benthem, Rom JM Perenboom, M Elske van den Akker-van Marle, Barbara S Niël-Weise

**Affiliations:** 1 Leiden University Medical Centre, Department of Infectious Diseases, Leiden, The Netherlands; 2 Dutch Institute for Healthcare Improvement, Utrecht, The Netherlands; 3 National Institute for Public Health and the Environment. Centre for Infectious Disease Control, Bilthoven, The Netherlands; 4 TNO Quality of Life. Department Innovation in Healthcare, Leiden, The Netherlands; 5 Leiden University Medical Centre, Department of Medical Decision Making, Leiden, The Netherlands; 6 Working Party Infection Prevention, Leiden, The Netherlands

## Abstract

**Background:**

Indwelling urinary catheters are the main cause of healthcare-associated urinary tract infections. It can be expected that reduction of the use of urinary catheters will lead to decreased numbers of urinary tract infection.

**Methods:**

The efficacy of an intervention programme to improve adherence to recommendations to reduce the use of urethral catheters was studied in a before-after comparison in ten Dutch hospitals. The programme detected barriers and facilitators and each individual facility was supported with developing their own intervention strategy. Outcome was evaluated by the prevalence of catheters, alternatives such as diapers, numbers of urinary tract infections, the percentage of correct indications and the duration of catheterization. The costs of the implementation as well as the catheterization were evaluated.

**Results:**

Of a population of 16,495 hospitalized patients 3335 patients of whom 2943 were evaluable for the study, had a urethral catheter. The prevalence of urethral catheters decreased insignificantly in neurology (OR 0.93; 95% CI 0.77 - 1.13) and internal medicine wards (OR 0.97; 95% CI 0.83 - 1.13), decreased significantly in surgical wards (OR 0.84; 95% CI 0.75 - 0.96), but increased significantly in intensive care (IC) and coronary care (CC) units (OR 1.48; 95% CI 1.01 - 2.17). The use of alternatives was limited and remained so after the intervention. Duration of catheterization decreased insignificantly in IC/CC units (ratio after/before 0.95; 95% CI 0.78 - 1.16) and neurology (ratio 0.97; 95% CI 0.80 - 1.18) and significantly in internal medicine (ratio 0.81; 95% CI 0.69 - 0.96) and surgery wards (ratio 0.80; 95% CI 0.71 - 0.90). The percentage of correct indications on the day of inclusion increased from 50 to 67% (p < 0.0001). The prevalence of urinary tract infections in catheterized patients did not change. The mean cost saved per 100 patients was € 537.

**Conclusion:**

Targeted implementation of recommendations from an existing guideline can lead to better adherence and cost savings. Especially, hospitals which use a lot of urethral catheters or where catheterization is prolonged, can expect important improvements.

## Background

In 1927 the indwelling urinary catheter was introduced by F. E. B. Foley [1]. In the beginning the catheters drained into an open bucket; in the sixties of the previous century the closed drainage system was introduced. Urinary tract infections were substantially reduced by this change [2,3]. Ever since, prevention of urinary tract infections has been a major focus of hospital infection control. The present situation, at least in the Netherlands, is that all hospitals have guidelines and protocols to reduce urinary tract infections in patients with indwelling catheters. The latest version of the national guideline for prevention of urinary tract infections in patients with indwelling urinary catheters published by the Dutch Working Party on Infection Prevention (WIP) dates from 2005 [4]. The guidelines of the WIP are the national standards of infection prevention and local protocols are based on these guidelines.

Given the fact that the prevention of infections in patients with urethral catheters has already received considerable attention and the latest guideline does not offer new methods of prevention, the question is whether implementation activities can nevertheless improve the prudent use of urethral catheters. Recent intervention studies suggest that this is indeed the case [5-9].

We studied the efficacy of an implementation strategy focused on a limited number of recommendations from the WIP guideline, all aimed at reduction of the use of urethral catheters. Reduction means that fewer patients get a catheter and patients who need a catheter will have the catheter for a shorter period. We choose these recommendations because the use of a urinary catheter and the duration of catheterization are risk factors directly linked to the pathogenesis of urinary tract infections.

## Methods

### Study design

The study was set up as a before-after comparison consisting of three periods, before, during and after intervention. Ten Dutch hospitals scattered over the country participated. Hospitals involved in the Dutch surveillance programme of nosocomial infections (PREZIES) were approached with information about the project. Hospitals that responded positively were included. Out of the10 hospitals selected two were university hospitals located in Leiden and Amsterdam. The other hospitals were general acute care hospitals located in Veghel/Oss, Deventer, The Hague, Haarlem, Roermond, Rotterdam, Tiel and Tilburg.

The hospitals were randomized into group A and B. A piece of paper with the name of the hospital was put in an opaque envelope and sealed after which the envelopes were mixed. Someone not involved in the study was asked to put an A or B randomly on five envelopes, respectively. Group B acted as control for unintended changes in the outcome parameters during the intervention period in group A hospitals. Each hospital selected as many wards as needed so that the expected number of patients with a urethral catheter was approximately 20 per site visit. There was no restriction on the type of department. The only criterion for ward selection was the number of patients with catheters. Assuming a base line catheter prevalence of 30% and a minimal of four site visits per period per group of hospitals, and taking into account an expected maximal inter-hospital variation of 30%, the power to detect changes of 10% is expected to be 85%, to detect changes of 20% is 94% and to detect changes of 30% is 99%, when a number of 2000 patients per group is included. The study was assessed by the medical ethical committee of the Leiden University Medical Centre which concluded that the study focused on improvement of patient care and, therefore, did not require permission by the committee (CME 05/36).

### Intervention strategy

After a three-month period of baseline measurements the five hospitals allocated to group A started preparations for the intervention period in month four of the study. First in each hospital a small intervention group consisting of infection control professionals, (head)nurses, incontinence nurses and doctors, prepared the intervention, and then introduced it in the wards. In month nine the intervention period was closed. The hospitals of group B started the preparations in month nine after eight months of baseline measurements and stopped the intervention activities in month 14.

The aim of the intervention groups was to develop a method of implementation that best suited the specific circumstances of the hospital. The intervention groups were supported by a document describing intervention methods, literature about reducing the use of urinary catheters and a summary of the results of semi-structured group interviews in three hospitals to detect barriers and facilitators to the implementation. In one session per hospital an implementation expert instructed the local intervention group.

### Data collection

During a period of 17 months each hospital was visited 20 times to include patients and collect data. On a study day a research nurse visited the participating wards of a hospital and included all patients with a urinary catheter with the exception of those having catheters for urological interventions. For each patient age, sex, the day of insertion of the catheter, the indication for catheterization on the day of insertion and the study day, signs and symptoms of urinary tract infection, and antibiotic use were recorded. A urine sample was collected for laboratory tests. The numbers of patients admitted to a ward and of those using an alternative for bladder catheterization (e.g. diaper, condom catheter) were registered. The infection control professionals of the hospitals followed the patients until removal of the catheter or discharge from the ward to determine the number of catheterization days.

### Outcome parameters

The effect of the intervention was monitored by measuring the prevalence of patients with a urethral catheter, patients with an alternative to urethral catheterization, and urinary tract infections in catheterized patients, the percentage of patients with a correct indication for urethral catheterization at the time of catheterization and upon inclusion in the study, and duration of catheterization from insertion to removal.

Bacteriuria was defined as ≥ 10^5 ^colony-forming units/ml in at least one of the three media of a uricult (Uricult Plus; Orion Diagnostica, Finland). A symptomatic urinary tract infection was defined as the presence of fever or pus draining along the outside of the catheter or pain in the bladder region and bacteriuria. Symptomatic infection was also diagnosed when two of the above-mentioned clinical signs were present and the leukocyte esterase or nitrite test (Combur 2 Test^® ^strips, Roche Diagnostics) was positive. Asymptomatic urinary tract infection was defined as bacteriuria in the absence of any of the above-mentioned clinical signs [10].

Correct indication for urethral catheterization was defined according to generally accepted criteria [11,12].

### Cost evaluation

To calculate the costs of the implementation activities everyone involved recorded the time spent on the activities. Other costs such as travelling, meetings and material expenses were listed. Time was related to the gross salary of the different employees [13]. The price level of 2008 was applied.

The costs of catheterization and daily care for a patient with an indwelling catheter were calculated based on the costs of materials and the mean time spent by healthcare workers. Mean time was determined by asking 18 experienced nurses employed in 5 different hospitals to indicate the time needed for specific actions such as insertion and removal of the catheter, emptying and changing catheter bags, daily care of the catheter site and solving problems. Costs of materials were based on hospital purchase prices.

### Statistical analysis

For the analysis the period before intervention (start of study until first intervention activities in the wards) was compared with the period after intervention (start of activities in the wards until the end of the study). Time series analyses were used to estimate trends in outcome measures during the periods. The effect of the intervention on catheter prevalence and catheter duration was quantified by regression modeling. To account for paired observations, i.e. before and after intervention per hospital, hospitals were put in the model as random intercept. Intervention, ward type and their interaction were put in the model as fixed effects. The prevalence was modeled by a logistic regression model [14], while the duration was modeled by an exponential regression model [15]. For the latter, it was assumed that the probability of catheter removal is constant over time. Censoring was allowed. The models were fit using the statistical software package R [16]

The number of catheter days eliminated by the intervention was calculated by subtracting the average number of catheter days before and after the intervention, respectively, per hospital. All these figures were added to calculate the total number of saved catheter days per 100 patients.

## Results

### Hospitals and patients

The hospitals were visited a total of 791 times; 16,495 patients were admitted to the participating wards. The number of patients with a urethral catheter was 3335. Of them 392 patients could not be included, because they were too ill, were nursed in isolation or temporarily absent for examinations or surgery. Of the included patients 483 (16%) were admitted to the intensive care or coronary care unit, 652 (22%) to an internal medicine ward, 1325 (45%) to a surgery ward and 483 (16%) to a neurology ward.

Since time series analysis revealed no significant trends in outcome measures over time before the intervention (data not shown), data from each hospital gathered during the multiple visits before the start of the intervention were combined. The same was the case for data gathered during the visits after the intervention (data not shown). Excluding visits made during the 4-month period of actual intervention activities in the hospitals did not change the results (data not shown). Data from group A and B were merged because no significant differences were found between the groups (data not shown).

### Interventions

Each hospital developed its own implementation strategy (table 1). Looking back the activities can be categorized into three types. The first is revision of existing protocols and materials used for catheterization, as was done by seven hospitals. The second category is education and providing information by oral (9 hospitals) or written (8 hospitals) instructions, by posters (6 hospitals), pocket cards (3 hospitals) or holding a competition (1 hospital). The third category is changing daily practice by paying attention explicitly to catheterized patients during the daily work meetings (6 hospitals), putting a reminder on the patient's record (4 hospitals), a fixed stop order for removal of the catheter (1 hospital), introducing a bladder scan (2 hospitals) or involving a specially trained nurse (2 hospitals).

**Table 1:** Intervention activities per hospital

Hospital	Revision of protocols and materials	Education and information	Changing daily practice
A1		Written information Oral information Poster Competition	Explicit attention during daily rounds

A2	Protocol revision	Oral information	Reminder on patient record Fixed stop order for removal catheter Explicit attention during daily rounds Introduction of bladder scan

A3		Written information Oral information	Explicit attention during daily rounds Involving specially trained nurses Introduction of bladder scan

A4	Protocol revision	Oral information Poster	Reminder on patient record Explicit attention during daily rounds

A5	Protocol revision	Written information Oral information	Involving incontinence nurse

B6	Protocol revision	Written information Oral information Poster Pocket card	

B7	Protocol revision Materials revision	Written information Oral information Pocket card	Reminder on patient record Explicit attention during daily rounds

B8	Protocol revision	Written information Oral information Poster Pocket card	

B9	Protocol revision	Written information Poster	Explicit attention during daily rounds

B10		Written information Oral information Poster	Reminder on patient record

### Urethral catheters

Before the implementation activities on the wards started there were 1149 catheterized patients. After the interventions had been started there were 1794 patients.

The average prevalences of urethral catheters before and after the interventions are shown in table 2 for each hospital per ward type. For intensive care and coronary care units the prevalence increased after intervention. On the neurology and internal medicine wards the prevalence decreased although it was not statistically significant. The largest, statistically significant decrease was observed for the surgery wards (table 3).

**Table 2:** Average catheter prevalence (%) per ward type before and after intervention.

Hospital	Admitted patients (N)		ICU/CCU		Internal medicine		Neurology		Surgery	
	**before**	**after**	**before** **(155)^1)^**	**after** **(328)^1)^**	**before** **(274)^1)^**	**after** **(378)^1)^**	**before** **(162)^1)^**	**after** **(321)^1)^**	**before** **(558)^1)^**	**after** **(767)^1)^**

A1	426	958	-	-	14	16	25	18	22	21

A2	452	619	85	84	10	22	19	22	16	14

A3	345	1434	82	97	8	15	19	25	32	22

A4	221	1205	88	87	21	14	28	15	23	13

A5	290	802	-	-	16	16	20	18	34	28

B6	1208	1834	57	66	14	12	15	13	20	21

B7	520	623	-	-	20	11	21	21	-	-

B8	1573	720	-	-	13	12	-	-	23	17

B9	420	546	63	60	16	16	-	-	15	16

B10	949	1350	-	-	14	15	-	-	17	18

Total	6404	10,091	74	81	14	14	20	19	21	19

ICU = intensive care unit. CCU = coronary care unit

1) Number of catheterized patients

**Table 3:** Comparison of catheter prevalence and duration of catheter before and after intervention.

	Catheter prevalence	Duration of catheter
	**Odds ratio** **(95% confidence interval)**	**Ratio after/before** **(95% confidence interval)**

Intensive care and coronary care units	1.48 (1.01 - 2.17)	0.95 (0.78 - 1.16)

Internal medicine	0.93 (0.77 - 1.13)	0.97 (0.80 - 1.18)

Neurology	0.97 (0.83 - 1.13)	0.81 (0.69 - 0.96)

Surgery	0.84 (0.75 - 0.96)	0.80 (0.71 - 0.90)

### Alternatives for urethral catheterization

The use of alternatives for urethral catheterization such as condom catheters and diapers fluctuated considerably. The prevalence was 12.5% (95% CI 11.7 - 13.3) before and 11% (95% CI 10.4 - 11.6) after the interventions.

### Duration of catheterization

The average durations of catheterization before and after the interventions are shown in table 4 for each hospital per ward type. The average duration of catheterization decreased for all ward types. On intensive care and coronary care units and neurology wards the decreases were small and statistically insignificant. Larger and statistically significant decreases were seen on the internal medicine wards and surgery wards (table 3). In total, per 100 catheterized patients 460 (95% CI 162 - 761) catheter days were eliminated by the intervention.

**Table 4:** Average duration of catheter in days per ward type before and after intervention.

Hospital	ICU/CCU		Internal medicine		Neurology		Surgery	
	**before**	**after**	**before**	**after**	**before**	**after**	**before**	**after**

A1	-	-	9.3	14.7	9.7	11.2	12.6	16.3

A2	6.7	11.6	-	-	15.8	20.5	9.9	8.8

A3	70.0	29.6	7.0	14.0	41.3	16.7	13.2	10.3

A4	34.5	12.5	-	-	9.5	9.7	6.6	6.8

A5	-	-	21.5	10.1	8.8	12.0	18.4	10.8

B6	30.6	14.6	20.3	12.8	13.9	15.2	8.9	8.5

B7	-	-	14.9	12.4	16.5	17.3	16.8	9.3

B8	-	-	13.0	10.6	-	-	14.3	9.6

B9	24.4	10.0	14.1	12.5	-	-	11.4	15.9

B10	-	-	7.4	12.3	-	-	10.9	8.9

Total	25.6	16.2	14.9	12.5	15.8	15.7	11.8	10.5

ICU = intensive care unit. CCU = coronary care unit

### Indications for catheterization

Surgery and monitoring of urine production were the most common indications for catheterization; 60% on the day of insertion and 50% on the day of inclusion in the study. The third most frequent category was unknown reason for catheterization. On the day of insertion this was the case for one-fifth of the catheters and on the study day for almost two-fifth of the cases. Before the intervention 64% of the catheters were inserted for a correct reason, on the day of inclusion this was the case for 50%. After the intervention the percentage of correct indications increased to 74% on the day of insertion and 67% on the day of inclusion (p < 0.0001).

### Urinary tract infections

Clinical signs and symptoms observed among the patients were fever (27%), pus along the catheter (3%), pain in the bladder region (5%), abdominal pain (2%), and pain in the renal region (0.5%). Symptomatic urinary tract infection was present in 12.6% of the patients before the intervention and in 12.7% after the intervention. Ninety-five percent of the patients with symptomatic urinary tract infections had bacteriuria besides symptoms. Asymptomatic urinary tract infection was diagnosed in 37.4% of the patients before intervention and in 38.3% after intervention.

### Costs

The mean costs of the implementation programme per hospital were € 2638, including the implementation expert, and € 1993 without the implementation expert. The costs per hospital varied from € 1023 to € 3763.

The costs of insertion of an indwelling catheter were calculated as € 28, removal of the catheter as € 3 and daily care as € 3. Costs of catheterization per 100 hospitalized patients are summarized in Table 5. In eight of the 10 hospitals costs of bladder catheterization per 100 patients were less after the intervention than before. The mean amount saved was € 537 per 100 hospitalized patients.

**Table 5:** Catheterization costs

Hospital	Catheterization costs per 100 patients (€)		
	**Before**	**After**	**Difference**

A1	2442.30	1469.09	973.22

A2	1761.44	2129.56	-368.12

A3	2850.13	2267.46	582.67

A4	3530.07	1863.95	1666.13

A5	2364.93	1531.36	833.57

B6	1938.69	1498.85	439.84

B7	2415.56	1549.89	865.67

B8	1337.21	960.94	376.27

B9	1850.31	1650.25	200.06

B10	1112.74	1250.22	-137.49

Total	1862.67	1643.30	228.36

Mean	2138.88	1602.14	536.74

## Discussion

The central question of the present study was whether it is possible to reduce the use of indwelling bladder catheters in number and duration in hospitals that have followed a guideline to prevent urinary tract infections for many years. The results differed between medical disciplines. In surgical wards a significant decrease in the prevalence of urethral catheters and duration of catheterization was achieved. In internal medicine wards duration of catheterization decreased significantly. On inspection of data from the individual hospitals and wards it is striking that the largest effects of the interventions were found for wards with initially high rates of urethral catheters or long duration of catheterization. The efficacy of the intervention seems to relate to the point of departure of the hospital or ward. This observation leads to the recommendation that hospitals should perform surveillance of urethral catheters and duration of catheterization and should undertake action when the numbers are high. Reference data are needed for this purpose. National surveillance programmes such as the American NHSN (former NNIS), German KISS and Dutch PREZIES are best equipped to provide these figures.

A mixed effect regression modeling approach was used. An advantage of this approach over a hospital specific approach is that it allows for variation between hospitals, or correlation within hospitals. The estimated coefficients then apply to an individual (mean) hospital. For binomial data, such as prevalence, this is a commonly used approach; however, for censored duration data it is not. We therefore used the exponential model for duration whereby it is assumed that the probability of catheter removal at any time is constant over time. Random effects are incorporated in this model.

Indications for catheterization improved considerably on the day of catheter insertion and on the day of inclusion in the study. The number of catheters with unknown indication decreased by 40%. The percentages of incorrect indications on insertion (36%) and on the day of inclusion (50%) during the pre-intervention phase of our study are in line with the 21 to 64% of incorrect indications reported in literature [11,12,17,18]. We used generally accepted criteria to classify catheters as correctly or incorrectly indicated. Incorrect indications were monitoring of urine production in patients who can micturate on request and incontinence of urine unless open perineal or sacral wounds are present or patients are immobile with enhanced risk of getting bed sores. All other indications were considered correct. The question is whether the observed improvement in indications for catheterization means a real change in indications for catheterization or is due to better knowledge of the indications and, therefore, better formulation of the indications in the records.

The intervention programme led to cost savings in eight of the ten participating hospitals, easily compensating for the costs of the interventions. In the year 2006 Dutch hospitals admitted 1.7 million patients. This means that this implementation strategy for our national guideline in all Dutch hospitals about € 9 million could be saved per year. This figure may be a moderate overestimation of cost savings because we did not take into account nursing time involved with helping non-catheterized patients to go to the toilet or changing bed linen when a patient is incontinent for urine. In the setting of the acute care hospitals participating in the study we expect this to be the case in only a negligible number of patients.

The intervention programme did not result in a decrease in the number of patients with bacteriuria. Shortening the duration of catheterization could have this effect because there is a direct relationship between duration of catheterization and the occurrence of infection. Apparently, the reduction in catheterization time that was realized in our study was too small to lead to a reduction in bacteriuria. The same was observed by Loeb et al. [9] with much shorter catheter durations of 3 to 5 days than in our study in which after intervention duration varied between 10 to 16 days.

From the results of the study it cannot be deduced whether specific interventions used in the hospitals are effective or more effective than others. The study shows the effect of an implementation programme, implying detection of barriers and facilitators, and support of hospital teams by instruction and written information about developing interventions. This approach leads to hospital-tailored projects to improve patient care.

## Conclusion

Our study has shown that targeted implementation of recommendations from an existing guideline can lead to better adherence to the guideline and cost savings. Especially, hospitals that use many urethral catheters or have a long period of catheterization can expect important improvements by an intervention to reduce the use of urethral catheters.

## Competing interests

The authors declare that they have no competing interests.

## Authors' contributions

All authors read and approved the final manuscript. PJvdeB designed the study and was principal investigator. JCW contributed to the study design and was involved in the selection of participating hospitals. BHBvB contributed to the study design and did the data analysis. RJMP contributed to the study design and supervised the implementation programme. MEvdA-vM contributed to the study design and did the economic analysis. BSN-W contributed to the study design and did the literature analysis.

## Pre-publication history

The pre-publication history for this paper can be accessed here:

http://www.biomedcentral.com/1471-2490/11/10/prepub
